# Exacerbation of CNS inflammation and neurodegeneration by systemic LPS treatment is independent of circulating IL-1β and IL-6

**DOI:** 10.1186/1742-2094-8-50

**Published:** 2011-05-17

**Authors:** Carol L Murray, Donal T Skelly, Colm Cunningham

**Affiliations:** 1 School of Biochemistry and Immunology, Trinity College Institute of Neuroscience, Trinity College Dublin, Dublin 2, Rep. of Ireland

**Keywords:** microglia, priming, dexamethasone, chronic, neurodegeneration, systemic, inflammation, Alzheimer's disease, IL-1β

## Abstract

**Background:**

Chronic neurodegeneration comprises an inflammatory response but its contribution to the progression of disease remains unclear. We have previously shown that microglial cells are primed by chronic neurodegeneration, induced by the ME7 strain of prion disease, to synthesize limited pro-inflammatory cytokines but to produce exaggerated responses to subsequent systemic inflammatory insults. The consequences of this primed response include exaggerated hypothermic and sickness behavioural responses, acute neuronal death and accelerated progression of disease. Here we investigated whether inhibition of systemic cytokine synthesis using the anti-inflammatory steroid dexamethasone-21-phosphate was sufficient to block any or all of these responses.

**Methods:**

ME7 animals, at 18-19 weeks post-inoculation, were challenged with LPS (500 μg/kg) in the presence or absence of dexamethasone-21-phosphate (2 mg/kg) and effects on core-body temperature and systemic and CNS cytokine production and apoptosis were examined.

**Results:**

LPS induced hypothermia and decreased exploratory activity. Dexamethasone-21-phosphate prevented this hypothermia, markedly suppressed systemic IL-1β and IL-6 secretion but did not prevent decreased exploration. Furthermore, robust transcription of cytokine mRNA occurred in the hippocampus of both ME7 and NBH (normal brain homogenate) control animals despite the effective blocking of systemic cytokine synthesis. Microglia primed by neurodegeneration were not blocked from the robust synthesis of IL-1β protein and endothelial COX-2 was also robustly synthesized. We injected biotinylated LPS at 100 μg/kg and even at this lower dose this could be detected in blood plasma. Apoptosis was acutely induced by LPS, despite the inhibition of the systemic cytokine response.

**Conclusions:**

These data suggest that LPS can directly activate the brain endothelium even at relatively low doses, obviating the need for systemic cytokine stimulation to transduce systemic inflammatory signals into the brain or to exacerbate existing pathology.

## Background

It is known that chronic neurodegeneration is associated with an inflammatory response, chiefly mediated by the brain macrophage population, the microglia. However it remains unclear how this microglial response contributes to neurodegeneration and, thus far, non-steroidal anti-inflammatory drugs (NSAIDs) have not proved helpful in patients with dementia [1]. We have previously shown that the microglial response in the ME7 model of prion disease is characterised by a muted inflammatory phenotype with very limited synthesis of pro-inflammatory cytokines [2,3]. However these microglia are primed by disease to respond more robustly to subsequent inflammatory challenges either peripherally or centrally [4]. One consequence of this is that animals with existing disease show exaggerated sickness behaviour responses to systemic administration of bacterial endotoxin lipopolysaccharide (LPS), used to mimic systemic infection in these animals [5,6]. Furthermore, we have shown that cognitive dysfunction [7] and neuronal death [4] can both be acutely exacerbated by the superimposition of systemic inflammatory challenge on a background on progressing disease and that disease progression itself can be expedited following systemic inflammatory challenge with LPS [6] or poly I:C (double stranded RNA analogue used to mimic systemic viral infection) [8]. There is now growing evidence from animal models of Alzheimer's disease [9-11], Parkinson's disease [12,13], aging [14,15], amyotrophic lateral sclerosis [16], stroke [17], experimental autoimmune encephalomyelitis [18] and Wallerian degeneration [19] that these exacerbating effects of systemic inflammation occur not only in the ME7 model of prion disease but constitute a generic phenomenon whereby systemic inflammation has more severe outcomes in individuals with prior pathology in the CNS (see [20] for review). Importantly, this phenomenon also appears to occur in the human population: we have now shown in an Alzheimer's disease patient cohort that systemic inflammatory events are associated with more rapid cognitive decline [21]. Clinically it is well recognised that patients with dementia frequently deteriorate after systemic infections, suffering episodes of delirium and long-term cognitive impairment, but this area remains little studied. Thus, it is now a priority to address mechanisms by which systemic inflammation may bring about these deleterious changes in those brains with pre-existing pathology.

In the current study we addressed the hypothesis that systemic cytokines play a role in this exacerbation of CNS inflammation, heightened sickness behaviour responses and the associated increase in apoptosis in the CNS. We have inoculated animals with the ME7 strain of prion disease or with normal brain homogenate (NBH) and then challenged these animals with LPS (500 μg/kg) in the presence or absence of the synthetic glucocorticoid dexamethasone-21-phosphate to inhibit cytokine synthesis induced by LPS. These animals were assessed on measures of sickness behaviour, systemic and CNS induction of cytokines and on induction of new apoptotic events. We found that while dexamethasone-21-phosphate did inhibit systemic cytokine synthesis and did block the hypothermic response it had no significant effect on activity and did not block CNS synthesis of cytokine mRNA or microglial IL-1β nor prevent acute neurodegeneration induced by LPS.

## Methods

### Stereotaxic surgery, interperitoneal challenges and transcardial perfusion

All animal procedures were carried out under licence from the Department of Health and Children, Republic of Ireland or the UK Home Office after internal ethical review. Female C57BL/6 mice (Harlan, UK) were housed in groups of 5 on arrival, with food and water ad libitum. Females were used in order to avoid fighting. The holding room was held at 21 C with a 12:12 h light-dark cycle (lights on at 0700 h). For surgical inoculation with prion disease mice were anaesthetised with intra-peritoneal Avertin and positioned in a stereotaxic frame. 1 μl of a 10% w/v scrapie-infected (ME7 strain) C57BL/6 brain homogenate (or 10% w/v normal brain homogenate (NBH)) was injected into both dorsal hippocampi (from bregma: anterior-posterior -2.0 mm, lateral -1.7 mm, depth -1.6 mm) using a 10 μl Hamilton microsyringe. At 18-19 weeks post-inoculation with ME7 prion disease, further injections were made intraperintoneally (i.p.) as described: Dexamethasone-21-phosphate was prepared in sterile saline and administered at a concentration of 2 mg/kg 1 hour before LPS (*Salmonella Equine abortus*, Sigma, Dorset, UK), which was administered at a dose of 500 μg/kg. This dose was previously used to show phenotype switching of primed microglia and to cause acute neuronal death in ME7 animals [4]. Control animals were administered sterile saline. Animals were transcardially perfused at 4 hours to examine CNS cytokine transcription and blood levels of cytokines after assessment of sickness behaviour. Further animals were transcardially perfused with heparinized saline and 10% formalin at 3 hours or 15 hours to examine microglial IL-1β expression and apoptotic cell death respectively. Further animals were challenged with biotinylated LPS (100 μg/ml) as described below.

### Body temperature

Body temperature was measured using a rectal probe (TH-5 thermoprobe, Physitemp, NJ). This was done on 3 occasions in the week before LPS/dexamethasone injections to habituate mice to this procedure. Mice were then assessed at the time of the first injection and at 3.5 hours post-injection.

### Open field rears

Rears were assessed using activity monitor software (Med Associates Inc., Vermont, USA) as previously described [22]. The open field consisted of an aluminium base (27 × 27 cm) enclosed on four sides with 0.7 cm-thick clear acrylic sheet, surrounded by an opaque screen. Rears were measured the day before LPS challenge (baseline) to minimize habituation on the day of the LPS challenge and then at 2 hours following the i.p. challenges.

### ELISA for plasma cytokines

Under terminal anaesthesia, induced 4 hours after challenge with LPS, the thoracic cavity was opened and blood collected into heparinized tubes directly from the heart. This whole blood was centrifuged to remove cells and the remaining plasma aliquoted and stored at -20°C until assay. These samples were then analysed for IL-1β, TNF-α and IL-6. Samples were serially diluted and quantified only if the absorbance recorded fell on the linear portion of the standard curve. Mouse IL-6 and IL-1β were measured by ELISA using R&D systems duo set kits using a standard protocol. Briefly, the capture anti-IL-6 and IL-1β antibodies were all diluted 1/180 (approximately 1 μg/ml) in PBS and used to coat a 96 well maxisorb microplate overnight (Nunc; Fisher Scientific, Leicestershire) with 100 μl per well. Plates were then washed with PBS + 0.05% Tween and blocked with PBS + 1% BSA before addition of samples, serially diluted as before. Standards were prepared in the range 8-1000 pg/ml. Detection antibodies were used at 1/180 in 1% BSA/PBS and Streptavidin-poly horseradish peroxidase was diluted 1:10.000 in high performance ELISA buffer. TMB and H_2_O_2 _were used as substrate and the reaction was stopped with 1 M H_2_SO_4 _and the optical density read at 450 nm with correction at 570 nm. The reliable quantitation limit of all assays was 15.6 pg/ml.

### Taqman quantitative RT-PCR

Animals were terminally anaesthetised and transcardially perfused with heparinized saline (4 hours post-LPS). Brains were rapidly removed and a 2-3 mm thick coronal section was cut to allow the dissection of the areas containing the hippocampus. These tissues were placed in eppendorf tubes, snap frozen on liquid nitrogen and stored at -80°C until further use. All equipment and reagents were supplied by Applied Biosystems Ltd. (Warrington, UK) unless otherwise stated. Assays for IL-1β, IL-6, TNFα, and TGFβ1 were designed using the published sequences for these genes, applied to Primer Express™ software and have been previously published [23]. Where possible, probes were designed to cross an intron such that they were cDNA specific. For Taqman PCR, cDNA was generated from total RNA using Taqman Gold RT reagents. Two hundred ng of total RNA were reverse transcribed in a 10 μl reaction volume. One μl of the RT reaction (equivalent to 20 ng RNA) was subsequently used for the PCR. Cytokine and mRNA expression was assessed using relative quantification. Briefly, an intra-cerebral challenge with 2.5 μg LPS, known to up-regulate all target transcripts in mouse brain was performed and tissue harvested at 6 hours. Total RNA was isolated and 1 μg of this used to synthesize cDNA. A standard curve was made with serial one in four dilutions of this cDNA, with the undiluted standard being assigned an arbitrary value from which all other values followed. Plotting these values against the threshold cycle values (Ct) produced in the quantitative PCR reaction produced a linear standard curve from which relative concentration values were calculated from the Ct values of unknown samples. Thus all data for these quantifications are expressed as relative expression (arbitrary units). These data were normalised to the expression of the housekeeping gene glyceraldehyde-3-phosphate dehydrogenase (GAPDH), which was measured in each sample using Applied Biosystems Rodent GAPDH Taqman kit.

### Biotinylation of LPS

The protocol for biotinylation of LPS was adapted from that of Luk et al., [24] using EZ-link Biotin-PEG4-hydrazide (Pierce, Caramlington, UK) according to manufacturers instructions. LPS, at a concentration of 1 mg/ml in 0.1 M sodium acetate, was oxidised by addition of 10 mM Sodium metaperiodate at ratio of 1:1 in a light sensitive vessel for 30 min on ice. Oxidation was stopped by addition of glycerol (final concentration 50 mM) for 5 min. A 10 ml Zeba Desalt spin Column (7 K MWCO; Pierce, Caramlington, UK) was equilibrated with coupling buffer (PBS). To remove excess periodate and glycerol, the sample was placed on column, spun at 1000 x *g *for 2 min and eluted. One part prepared 50 mM Biotin-PEG_4_-Hydrazide solution (dissolved in dimethyl sulfoxide; Pierce, Caramlington, UK) was added to nine parts oxidised and buffer exchanged LPS and mixed for 2 hours at room temperature with gentle end to end rotation. The biotinylated molecules were then separated from non-reacted material by being spun at 1000*g *in a desalting column pre-equilibrated with coupling buffer (0.9% NaCl). Controls were prepared by mixing PBS for 2 hours with Biotin Hydrazide solution, followed by desalting as for LPS. Animals were then injected i.p. with 100 μg/kg of biotinylated LPS in a volume of 200 μl non-pyrogenic sterile saline. Control animals were administered normal LPS or PBS subjected to the biotinylation procedure.

### ELISA for Biotinylated LPS

Plasma levels of biotinylated LPS were assessed using an ELISA assay developed in-house. A 96-well maxisorb microplate (Nunc, Fisher Scientific, Leicestershire) was coated overnight with 100 μl per well of mouse monoclonal anti-biotin antibody (1 μg/ml; Invitrogen, Paisley, UK). The plate was washed 3 times with PBS + 0.05% Tween and blocked with PBS + 1% BSA before addition of 100 μl samples and standards. Samples were serially diluted 1/3, 1/9 and 1/27. Standards were prepared from a stock of biotinylated LPS in the range of 0-2000 pg/ml. The plate was washed again 3 times and 100 μl Streptavidin poly-horseradish peroxidise (poly-HRP: Sanquin, Amsterdam, Netherlands, diluted 1:10,000) was added to each well. Following another 3 washes, TMB and H_2_O_2 _were used as substrate and the reaction was stopped with 1 M H_2_SO_4 _before optical density was read at 450 nm with correction at 570 nm. Samples, standards, detection antibodies and Streptavidin poly-horseradish peroxidise were diluted in high performance ELISA buffer (Sanquin; Amsterdam, Netherlands). Sample concentrations were determined by extrapolation off the standard curve.

### Immunohistochemistry

Immunohistochemistry for IL-1β, IBA-1, cyclooxygenase-2 (COX2) was carried out on formalin-fixed, paraffin-embedded sections. Sections were de-waxed, re-hydrated and then quenched with 1% H_2_O_2 _in absolute Methanol for 20 min and washed briefly in PBS before antigen retrieval by microwaving in citrate buffer for 2 x 3 minutes with 5 minutes cooling in between incubations. Primary antibodies against COX-2 were obtained from Santa Cruz (CA, US), those against IL-1β from Peprotech (London, UK), those against IBA-1 from Abcam (Cambridge, UK). Biotinylated secondary antibodies, normal sera and avidin-biotin complex were from Vector Laboratories (Peterborough, UK). Avidin-horseradish peroxidase was obtained from DAKO (Cambridge, UK). Immunohistochemistry for all antigens was carried out using the Avidin-Biotin-Complex (ABC) method with minor modifications, depending on the antibody used, and has been described in detail in previous publications [7,25]. Labelling was visualised using the ABC method using peroxidase as enzyme, 0.015% v/v hydrogen peroxide as substrate and diaminobenzidine as chromagen.

### TUNEL

Immunohistochemistry for apoptotic cells was performed using the "Dead End" TUNEL staining kit (Promega, Southampton, UK). We have previously shown that while activated caspase-3 is more suitable for double staining to confirm the cell type, TUNEL is more suitable for quantification of cell death. Since we have previously demonstrated that the cell death is neuronal in this paradigm and the aim of the current study is to quantitatively assess whether dexamethasone-21-phosphate can protect against this apoptosis, TUNEL rather than caspase-3, was the appropriate strategy. Non-specific peroxidise activity was eliminated by incubating sections in 1% H_2_O_2 _for 10 min. Sections were washed in 0.85% NaCl for 5 min and PBS 2 × 3 min, before being pre-treated with proteinase K (10 or 20 μg/ml) for 5 min. After PBS 2 × 3 min wash, sections were preincubated with equilibration buffer for 10 min and then with TUNEL buffer (25 μl per section: 22 μl equilibration buffer, 2.5 μl nucleotide mix, 0.5 μl TdT enzyme) for 2 h at 37°C, labelling apoptotic cells with flourescein. The reaction was stopped using 2× sodium citrate solution to sections for 15 min. After 3 × 3 min PBS wash sections were blocked with 10% normal goat serum for 30 min and incubated with biotinylated anti-flourescein antibody (5 μg/ml) and continued with the normal Avidin-Biotin-Complex as described above. Cells that were TUNEL-positive and also showed evidence of nuclear condensation were counted in the hippocampal and thalamic regions of 10 μm coronal sections of ME7 animals treated with LPS in the presence or absence of dexamethasone-21-phosphate. Two sections were counted and summed for each animal and groups of n = 4 or n = 5 animals were grouped to calculate average numbers of cells per treatment.

### Statistics

Since temperature and activity data were expressed as deviation from or percentage of baseline, they were analysed by one-way ANOVA and, contingent on a significant main effect, were subjected to limited pair-wise comparisons by Bonferroni post-hoc tests, the comparisons performed have been specified in the results text where appropriate. All peripheral ELISA data, CNS cytokine transcription data and apoptotic cell counts were analysed by one-way ANOVA and by limited pair-wise Bonferroni post-hoc comparisons contingent on a significant main effect.

## Results

### Hypothermia and sickness behaviour

**Figure 1:**
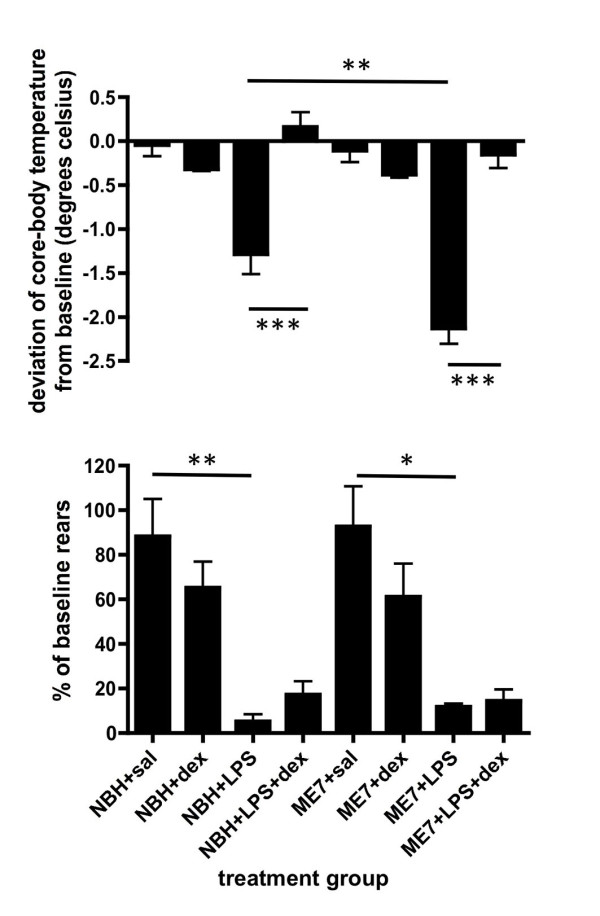
**Impact of dexamethasone-21-phosphate on LPS-induced core body temperature and activity changes**. a) The deviation of core body temperature, at 3.5 hours, from baseline (t = 0) in normal brain homogenate (NBH) or ME7 animals treated with saline or LPS (500 μg/kg), pre-treated with dexamethasone-21-phosphate (DEX; 2 mg/kg) or sterile saline. ** p < 0.01, *** p < 0.001 by Bonferroni post-hoc test after a significant main effect of treatment by one-way ANOVA. n = 9 (NBH+saline), n = 6 (NBH+LPS), n = 10 (ME7+saline, ME7+LPS), n = 8 (ME7+LPS+dex) and n = 5 for all other groups. b) Rearing activity at 2 hours post-challenge with LPS expressed as a percentage of baseline rears (-24 hours). *p < 0.05, ** p < 0.01 by Bonferroni post-hoc test after a significant main effect of treatment by one-way ANOVA. In assessment of rears n = 4 for all groups except ME7+LPS+dex (n = 5), NBH+saline (n = 10). All data have been presented as mean ± SEM.

Injection of LPS (500 μg/kg) in normal animals induced a clear hypothermic response with respect to NBH+saline animals while dexamethasone-21-phosphate appeared to have little effect (Figure 1a). Pre-treatment with dexamethasone-21-phosphate completely blocked the occurrence of this LPS-induced hypothermic response in normal animals (p < 0.001). When these experiments were repeated in ME7 animals the hypothermic response was significantly exaggerated in ME7+LPS animals compared to NBH+LPS (p < 0.01). It was apparent that despite the heightened hypothermic response in ME7+LPS animals, dexamethasone-21-phosphate was still capable of reversing this hypothermic response (p < 0.001). In addition, subjective assessment of these mice indicated that dexamethasone-21-phosphate significantly inhibited the appearance of sickness signs such as hunched posture and ruffled fur.

Rears were also very markedly affected by LPS treatment (figure 1b). LPS induced statistically significant decreases in both NBH and ME7 groups when compared to NBH+saline (p < 0.01) and ME7+saline groups (p < 0.05). The pre-treatment of animals with dexamethasone-21-phosphate before the LPS treatment did not prevent the marked decrease in rears in either diseased or normal animals. That is to say NBH+LPS were not statistically different to NBH+LPS+dex and ME7+LPS were not significantly different to ME7+LPS+dex (p > 0.05). Thus dexamethasone-21-phosphate does not protect against decreased locomotor activity despite protecting against the LPS-induced hypothermic response.

### Systemic cytokine response

**Figure 2:**
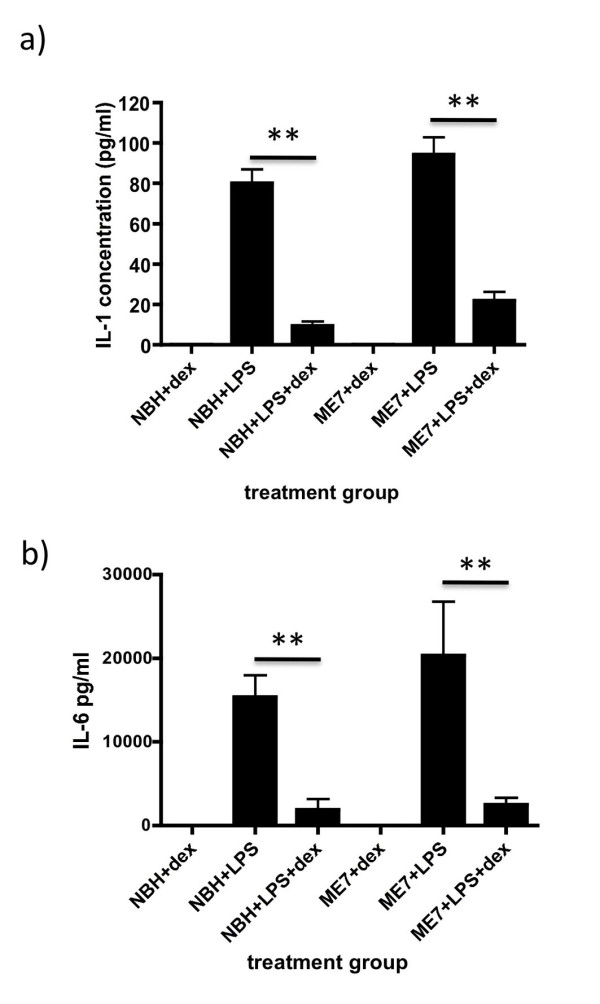
**Impact of dexamethasone-21-phosphate on LPS-induced systemic cytokine synthesis**. a) IL-1β and b) IL-6 concentrations, as measured by ELISA, in NBH and ME7 animals (18-19 weeks) at 4 hours post-LPS (500 μg/kg) with or without pre-treatment with dexamethasone-21-phosphate (2 mg/kg). ** p < 0.01, *** p < 0.001 by Bonferroni post-hoc test after a significant main effect of treatment by one-way ANOVA. n = 5 for NBH+LPS+dex and ME7+LPS+dex and n = 4 for all other groups. All data have been presented as mean ± SEM.

Plasma levels of IL-1β and IL-6 were assessed by ELISA assay at 4 hours post-challenge with LPS in the presence or absence of dexamethasone-21-phopshate. Levels of both cytokines were markedly increased after challenge with LPS and were very significantly diminished by pre-treatment with dexamethasone-21-phosphate. IL-1β was decreased by approximately 80% and this decrease was statistically significant as analysed by one-way ANOVA followed by Bonferroni post-hoc comparison of NBH+LPS versus NBH+LPS+DEX and of ME7+LPS versus ME7+LPS+DEX (both p < 0.001; figure 2a). IL-6 production was diminished by approximately 90% and once again this decrease was statistically significant for both NBH+LPS+DEX and ME7+LPS+DEX groups when compared to their respective control LPS-treated groups (p < 0.001; Figure 2b). TNF-α levels were also assessed, but these were below reliable quantification limits, as would be predicted at 4 hours post-LPS [7]. We have previously shown that dexamethasone at the current dose does block LPS-induced TNF-α [26]. Likewise, though NBH+saline and ME7+saline controls have not been included here, it is clear that saline does not induce these systemic cytokines in these animals [7].

### Hippocampal cytokine transcription

**Figure 3:**
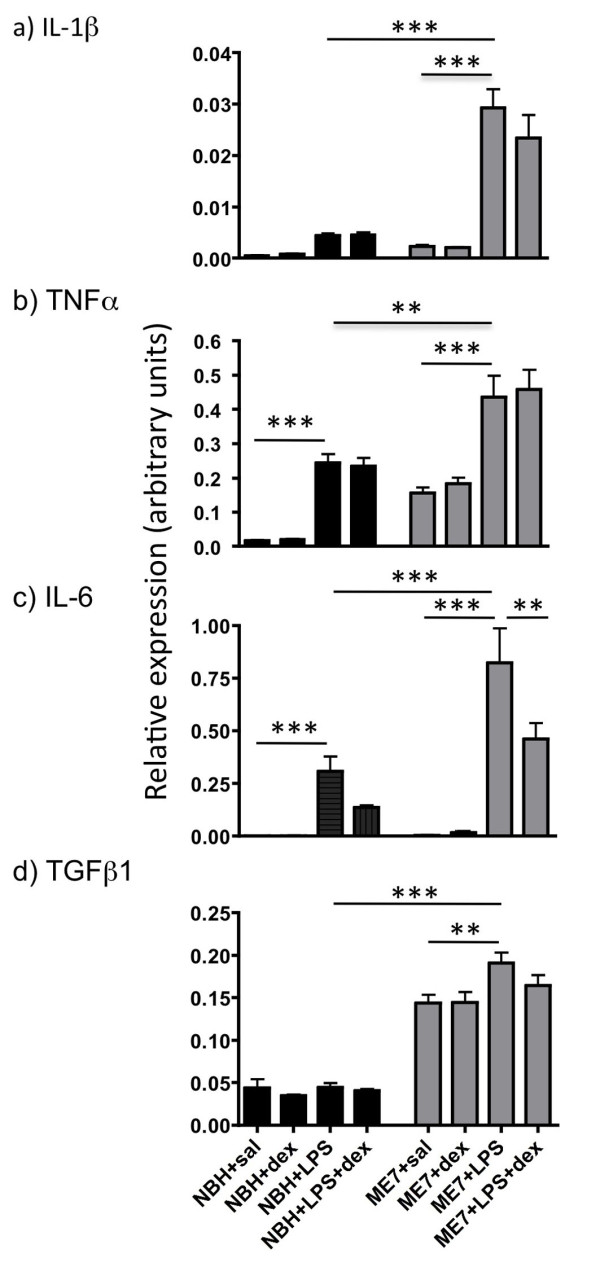
**Impact of dexamethasone-21-phosphate on LPS-induced CNS cytokine transcription**. TAQMAN quantitative PCR mRNA expression analysis of a) IL-1β, b) TNFα, c) IL-6 and d) TGFβ1. LPS (500 μg/kg) induced marked expression of all cytokines except TGFβ1 but dexamethasone-21-phosphate did not inhibit this transcription in most cases (p >> 0.05). Data were analysed by Bonferroni post-hoc test after a significant main effect of treatment by one-way ANOVA, ** p < 0.01, *** p < 0.001. n = 9 for ME7+saline, n = 4 for NBH+dex and NBH+LPS and n = 5 for all other groups. All data have been presented as mean ± SEM.

The transcription of mRNA for IL-1β, TNFα, IL-6 and TGFβ1 was assessed using quantitative PCR. These data have been assessed by one-way ANOVA, followed, if significant, by limited pair-wise comparisons using Bonferroni post-hoc tests. These data are shown in Figure 3.

Limited IL-1β was induced by LPS in NBH animals and indeed this increase did not reach statistical significance with respect to NBH+saline treated animals (p > 0.05, Figure 3a). The increase was much more marked in the case of ME7 animals treated with LPS compared to ME7+sal (p < 0.001) and to NBH+LPS (p < 0.001). However neither in the case of NBH+LPS nor ME7+LPS did dexamethasone-21-phosphate inhibit this CNS transcription of IL-1β. That is to say, ME7+LPS animals were not significantly different from ME7+LPS+dex (p > 0.05).

As previously described, TNFα was transcribed to a higher degree in ME7 than in NBH animals (Figure 3b). Furthermore, there were significant increases in TNFα mRNA when either NBH (p < 0.001) or ME7 (p < 0.001) animals were challenged with LPS. This LPS-induced increase was greater in ME7 animals (p < 0.01). However, dexamethasone-21-phosphate failed to inhibit this LPS-induced TNFα mRNA increase in either NBH (p > 0.05) or ME7 (p > 0.05) animals.

IL-6 mRNA levels were extremely low in saline or dexamethasone-21-phosphate treated groups whether in NBH or ME7 animals. Levels of mRNA for this cytokine were dramatically induced after LPS treatment and these increases were greater in ME7+LPS than NBH+LPS (p < 0.001, figure 3c). Dexamethasone-21-phosphate inhibited LPS-induced expression of IL-6 mRNA by approximately 50% in both NBH and ME7 animals but only in the latter case did this inhibition reach statistical significance (ME7+LPS > ME7+LPS+dex, p < 0.01).

TGFβ1 mRNA was elevated in ME7 animals with respect to NBH animals. LPS did not produce substantial further increases in this mRNA species, although in the case of ME7+LPS this minor increase was statistically significant (p < 0.01). Dexamethasone-21-phosphate did not have significant effects on CNS TGFβ1 transcription (p > 0.05). These data are shown in figure 3d.

### Immunohistochemistry

**Figure 4:**
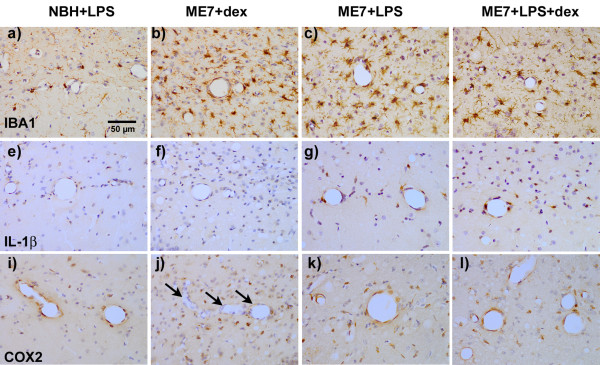
**Immunohistochemical detection of IBA-1, IL-1β and COX2 post-LPS**. a-d) IBA-1 labelling of microglia in normal (NBH) and ME7 animals treated with LPS (500 μg/kg) in the presence or absence of dexamethasone-21-phosphate (2 mg/kg) showing increased numbers and activated morphology in all ME7 groups. e-h) IL-1β labelling of microglial cells under the same conditions, showing robust cellular staining for IL-1β only in the ME7+LPS groups, in the presence or absence of dexamethasone. i-l) COX2 labelling of microglial and vascular endothelial cells in the same animals, showing induction of COX2 associated with the vasculature in all animals treated with LPS, but vessels remain unlabelled in ME7 animals treated only with dexamethasone-21-phosphate (arrows). Microglial cells are positively labelled with anti-COX2 antibodies in all groups, but these are more numerous in the ME7 animals. Magnification ×40, Scale bar = 50 μm in all photomicrographs.

IBA-1 labelling of microglia revealed that all three ME7 groups showed markedly increased microglial numbers and morphological evidence of activation (Figure 4a versus 4b, c and 4d). At 19 weeks post-inoculation with ME7 there remains significant inter-individual variation in the severity of microgliosis in the hippocampus. However, ME7 animals treated with dexamethasone-21-phosphate alone showed no evidence of IL-1β synthesis (Figure 4f). Conversely ME7 animals treated with LPS at 500 μg/kg showed clear labelling with IL-1β in microglial cells in the vicinity of the hippocampal blood vessels (Figure 4g) while normal animals treated with LPS at the same dose produced barely perceptible vascular-associated IL-1β (Figure 4e). The systemic pre-treatment with dexamethasone-21-phosphate did not block IL-1β synthesis in ME7+LPS animals (Figure 4h). The systemic challenge with LPS also produced clear expression of cyclo-oxygenase 2 in the vascular endothelium in both NBH and ME7 animals (Figures 4i and 4k respectively). It is clear that such vascular activation is not present in the absence of LPS (arrows, Figure 4j). Once again, inhibition of systemic cytokine synthesis using dexamethasone-21-phosphate did not block this endothelial activation of COX-2. Therefore, while dexamethasone-21-phosphate did substantially block systemic cytokines, this was insufficient to block CNS endothelial and microglial activation.

### Circulating LPS

Since the ablation of the systemic cytokine response clearly did not impact on CNS cytokine transcription of pro-inflammatory genes we investigated the possibility that LPS reaches the blood at measureable concentrations. We biotinylated LPS and injected mice with a concentration of 100 μg LPS/kg body weight. Even at this lower concentration this LPS was readily detectable by a novel ELISA assay using 96 well plates, coated with an anti-biotin antibody. Biotinylated LPS was detected at 30, 45 and 60 minutes post-i.p. injection demonstrating that LPS is in a position to activate the brain endothelium independent of circulating cytokines. Levels of biotinylated LPS were not significantly different at the different time points (30, 45 and 60 minutes) and were thus combined for the purposes of quantification. Biotinylated LPS was detected at an average level of 8.3 ± 1.8 ng LPS/ml of plasma. No signal was detected, using this ELISA, for non-biotinylated LPS or for PBS that was exposed to a biotinylation protocol identical to that used for LPS. In these animals treated with biotinylated LPS, we also sought to detect this labelled LPS at the cerebral endothelium in the hippocampal area. However we failed to detect any biotinylated LPS either by using the ABC method to directly bind peroxidase to biotin at the endothelium or by using an anti-biotin antibody that would allow detection of this biotin followed by the amplification facilitated by the addition of a secondary antibody before conjugation of the peroxidase enzyme. Thus, if LPS is bound at the endothelium in these experiments, it is at a level that is below the detection limit of the methods employed. We have verified that the biotinylated LPS is active, and thus can bind to its receptor complex, by demonstrating induction of TNF-α in plasma in each sample in which LPS was detected in the blood (data not shown).

### Quantification of apoptotic cell death

**Figure 5:**
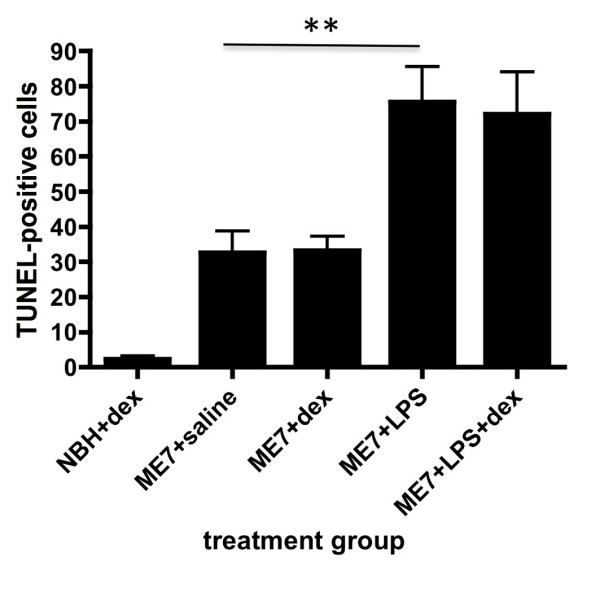
**TUNEL-positive apoptotic cells counts**. TUNEL-positive apoptotic cells were counted in the hippocampus and thalamus of 10 μm coronal sections from ME7 animals treated with or without LPS in the presence of absence of dexamethasone-21-phosphate. NBH animals treated with dexamethasone were also included to demonstrate reduced numbers in non-ME7 animals. Two coronal sections were counted and summed in each case. Data were analysed by Bonferroni post-hoc test after a significant main effect of treatment by one-way ANOVA, ** p < 0.01. n = 4 for ME7+dex and NBH+dex and n = 5 for all other groups. All data have been presented as mean ± SEM.

Apoptotic cell death was quantified at 15 hours post-challenge with LPS by counting the number of TUNEL-positive cells in coronal sections. These data are shown in figure 5. Evidence of nuclear condensation was also used to confirm apoptosis in cells that were TUNEL-positive. As previously described [4], there were very few if any TUNEL-positive cells detectable in NBH animals when compared to the relatively large numbers in ME7 animals at 18 weeks. There was a statistically significant increase in TUNEL-positive cells at 15 hours post-LPS (p < 0.01) but this increased apoptosis was not prevented by dexamethasone-21-pretreatment (p > 0.05) and dexamethasone alone did not induce apoptosis in ME7 animals.

### Hippocampal transcription of downstream effectors in ME7 animals

The transcription of mRNA for iNOS and COX2 was assessed using quantitative PCR. These data have been assessed by one-way ANOVA, followed, if significant by limited pair-wise comparisons using Bonferroni post-hoc tests. Limited iNOS mRNA was present in ME7 animals but this was significantly increased following LPS treatment (p < 0.001). Pre-treatment with dexamethasone-21-phosphate did not prevent this induction: ME7+LPS and ME7+LPS+dex were not significantly different.

**Figure 6:**
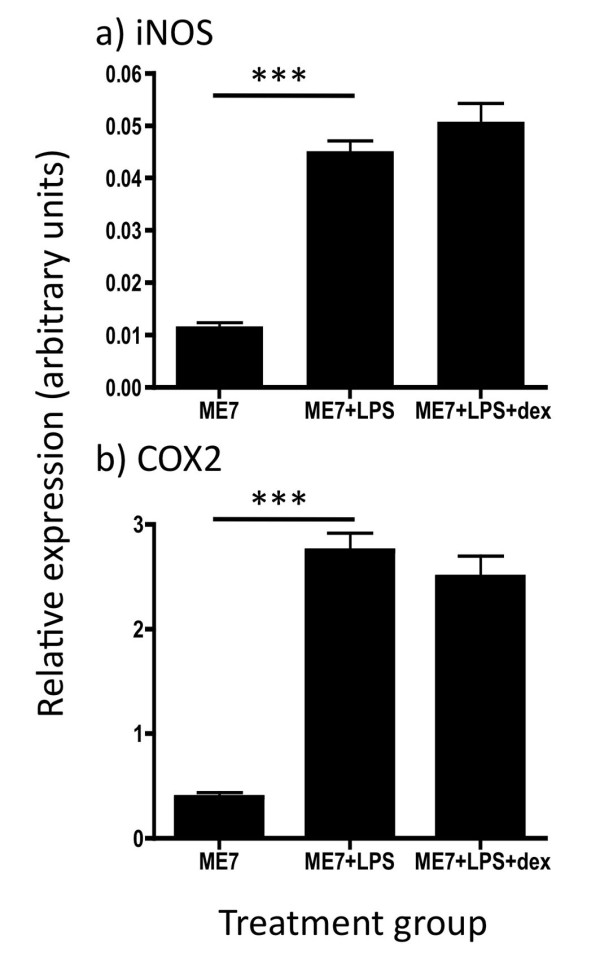
**Expression of iNOS and COX2 mRNA**. Transcription of the mRNA for secondary effectors iNOS and COX2 was assessed by quantitative PCR in the hippocampus of ME7 animals treated with saline or LPS (500 μg/kg), with the latter with or without pre-treatment with dexamethasone-21-phosphate (2 mg/kg). Data was analysed by one-way ANOVA with Bonferroni post-hoc pairwise comparisons. *** p < 0.001; n = 7 for ME7 and n = 5 for all other groups. All data have been presented as mean ± SEM.

COX2 mRNA appeared to be expressed at higher levels constitutively, consistent with the robust expression of COX2 in hippocampal neurons. Treatment with LPS produced a marked increase in its transcription compared to ME7 (p < 0.001). However dexamethasone-21-phosphate did not inhibit this CNS transcription of COX2. These data are shown in figure 6.

## Discussion

We have shown here that systemically administered dexamethasone-21-phopshate (2 mg/kg) is sufficient to block >80% of systemic LPS-induced IL-1β and IL-6. In addition this dose is sufficient to completely block the hypothermic response induced by LPS (500 μg/kg) in normal animals, and the exaggerated hypothermic response induced by LPS in ME7 animals. However, this dose could not block the LPS-induced decrease in open field rearing behaviour. Despite this robust inhibition of systemic inflammation, transcription of CNS cytokines occurs normally and microglia primed by prior neurodegeneration still effect robust synthesis of IL-1β protein. In addition, LPS-induced cell death, still occurs in the absence of significant systemic cytokines and in the presence of central IL-1β. Thus, LPS elicits an inflammatory response in the brain that is not inhibited by dexamethasone and is independent of its effect on circulating cytokines.

### Efficacy of dexamethasone-21-phosphate

The synthetic glucocorticoid dexamethasone-21-phosphate shows the classical steroidal anti-inflammatory effect in blocking systemic cytokine production to approximately 80-90%. This indicates that the pro-drug dexamethasone-21-phosphate has been converted to the active metabolite dexamethasone and has effected inhibition. Though the mechanisms of this inhibition are still controversial, it is widely accepted that dexamethasone can block NFκB-dependent gene transcription by direct transrepression [27] and the inhibition of cytokine synthesis observed in the current study is entirely consistent with this idea. However, the intact CNS transcription of cytokine genes that are also NFκB-dependent indicates that dexamethasone is either not effective in the CNS, or does not reach the CNS at sufficient concentrations to effect any inhibition of these processes. There is some evidence from prior studies of CNS injury that dexamethasone cannot block CNS cytokine transcription [28]. Dexamethasone is known to show much lower penetrance of the CNS than endogenous glucocorticoids [29] and those levels retained are significantly higher in the pituitary and the hypothalamus than in the hippocampus [30]. This appears to be controlled by the gene product of mdr1a (multiple drug resistance 1a), a drug transporting P-glycoprotein that functions as an extrusion pump to exclude xenobiotics from the brain parenchyma: brain dexamethasone concentrations are ten-fold higher in mdr1a-/- mice [31]. It thus seems likely that that dexamethasone does not penetrate the hippocampus at significant concentrations in the current study.

### Systemic cytokines are not necessary for sickness behaviour or CNS transcription

Despite its failure to effect inhibition of CNS cytokine transcription, dexamethasone was successful in this study in suppressing systemic cytokines IL-1β and IL-6 by approximately 80 and 90% respectively. This marked suppression of NFκB-dependent genes systemically was associated with a complete block of the LPS-induced hypothermic response. As previously described, this hypothermia was exaggerated in ME7 animals with respect to NBH animals given the same LPS challenge [5], but dexamethasone-21-phosphate was sufficient to block the hypothermic response in both groups. Both IL-1β and IL-6 have been strongly associated with the induction of fever responses in rodents in response to systemic LPS challenge. Systemic IL-1β is thought to be important in the induction of hypothalamic IL-6 and the subsequent induction of fever [32]. Hypothermia is known to occur in C57BL/6 mice when at ambient temperatures of 20-22°C [33] and while the molecular underpinning of this hypothermic response is less clear [34] it is nonetheless known that both systemic IL-1β and TNF-α can induce this response [35]. The current data are certainly consistent with the view that systemic cytokines are crucial in the hypothermic response since dexamethasone-21-phosphate prevents the hypothermic response in the current study. However, these cytokines are also described as being key mediators in the induction of sickness behaviour. In the current study it is notable that there is striking disconnection between the hypothermic response and the behavioural depression. Exploratory behaviour, as measured by rearing activity, is profoundly suppressed by LPS and this is not prevented by dexamethasone-21-phopshate, despite its marked effect in preventing systemic cytokine synthesis. These data are consistent with recent data showing that systemic administration of specific antibodies against the pro-inflammatory cytokines IL-1β, IL-6 and TNF-α fail to block LPS-induced inhibition of burrowing at low (1 μg/kg) and moderate (100 μg/kg) doses of LPS [36]. These systemically administered antibodies also failed to block CNS cytokine transcription [36]. In the current study, CNS cytokines were also not inhibited despite the effective blocking of systemic cytokine synthesis. Though we have not shown systemic TNF-α levels in the current study we did assess this: This was no longer detectable at 4 hours post-LPS. However, our previous studies have shown that TNF-α levels have returned to baseline 4 hours post-LPS [7] and that dexamethasone-21-phosphate, at the dose used in the current study, does inhibit systemic TNF-α synthesis [26]. Thus it is clear that robust systemic cytokine synthesis is not necessary for either CNS cytokine transcription (and translation) or for the expression of sickness behaviour.

### Alternative routes to CNS activation

The evidence that systemic cytokines can induce CNS cytokine transcription is substantial but it is increasingly clear, as discussed above, that they are not necessary for this induction. It is theoretically possible that LPS may activate the vagus directly. Though the NTS does not directly enervate the hippocampus, multisynaptic relays from the nucleus tractus solitarius (NTS) to the hippocampus have been identified [37]. However, we think that it is unlikely that vagal afferents have induced the neuroinflammation seen in presence of dexamethasone-21-phosphate in the present study. Vagal afferents also project, via the NTS, to the hypothalamus [38] but we see marked inhibition of hypothalamic activation in the presence of dexamethasone-21-phosphate (ie the hypothermic response induced by LPS was successfully blocked). An alternative mechanism by which CNS inflammation can be induced is via synthesis of prostaglandins. Our own studies previously showed that inhibition of COX1-mediated PGE2 production had no impact on LPS-induced brain cytokine transcription while inhibition of COX2-mediated PGE2 production, by nimesulide, provided partial inhibition of CNS IL-6 and TNF-α transcription [26]. However, neither the time course of COX-2 CNS transcription, nor that of systemic cytokines would appear to be sufficiently rapid to explain peak transcription of IL-1β and TNF-α occurring at one hour post-LPS in the normal brain [7]. A direct interaction between the LPS and the brain endothelium could explain this rapid CNS cytokine transcription. Recent data indicate that LPS can interact directly with brain endothelial cells *in vitro *[39] and the pattern and time course of gene induction in our own studies [7] are more consistent with LPS acting at the endothelium than with the actions of LPS-induced systemic cytokines, which first must be produced by tissue macrophages or circulating cells. In addition, Chakravarty & Herkenham showed that deletion of the TLR4 gene in peripheral cells was not sufficient to block CNS transcription of inflammatory genes, indicating that TLR4 expression at the brain endothelium was sufficient to effect blood to brain signalling [40]. Here we have labelled LPS using biotin and have clearly shown that LPS is present in the bloodstream as soon as 30 minutes after i.p. injection and thus would be present sufficiently early to induce the cytokine patterns observed here and in our earlier studies. Attempts to locate biotinylated LPS at the brain endothelium proved unsuccessful and although it was tempting to speculate that excessive biotinylation might prevent robust interaction between LPS and its receptor complex we also found that animals injected with the biotinylated form of LPS did produce a rapid systemic TNF-α response. Therefore the biotinylated LPS is clearly biologically active. Alternatively the failure to locate LPS at the endothelium may be explained by the low level expression of TLR4 and LPS binding proteins at the brain endothelium or indeed the relatively low levels of LPS in the circulation. Previous studies by Singh and Jiang have shown the presence of LPS lipids in endothelial preparations extracted from the CNS of rats treated systemically with LPS [39] and more recent studies by Banks et al., suggest that LPS minimally penetrates the brain but does show transient interaction with the endothelium [41].

Even with the demonstration, elsewhere, that LPS can act directly at the brain endothelium to induce CNS inflammatory gene transcription and with the rationale that dexamethasone-21-phosphate does not inhibit this transcription due to its exclusion from the brain parenchyma, this does not explain why COX-2 continues to be synthesized at the brain endothelium. Here we show clear evidence of mRNA transcription in the hippocampal homogenate, which therefore may represent induction of COX-2 in endothelial, microglial or neuronal cells, all of which we know to express COX-2. The immunhistochemistry on the other hand shows production of COX-2 protein, specifically in the endothelial layer. Thus we speculate on two possibilities to explain the surprising observation that COX-2 synthesis occurs at the endothelium despite dexamethasone inhibition. 1) Though it is known that COX-2 can be transcribed in an NFκB-dependent manner [42], there are multiple sites of regulation of its expression including cAMP (CREB), AP-1 and C/EBP [43] thus there may be NFκB-independent routes to its *de novo *transcription. Given the importance of signalling systemic inflammation to the brain it seems reasonable to predict a degree of compensation in the event of some routes of activation being blocked. 2) LPS interaction with its receptor complex might activate COX-2 protein translation from existing levels of endothelial mRNA transcripts: the immunohistochemical demonstration of COX-2 labelling in the current study is descriptive of the translation of mRNA to COX-2 protein and thus other mechanisms induced by endothelial LPS binding may be important in effecting this translation.

### CNS inflammatory effects of glucocorticoids

In a broader sense, it is also worthy of mention that there are a number of studies indicating that dexamethasone, glucocorticoids or behavioural stress can fail to block or can even exacerbate CNS NFκB activation [44] or inflammatory gene transcription [45] after systemic LPS treatment [46,47]. In an animal model of CNS neurotoxicity, systemic dexamethasone also exacerbated CNS cytokine transcription [28]. Thus glucocorticoids can exacerbate CNS inflammation rather than suppress it in certain situations (reviewed in [48]). The data in the current study argue that while CNS inflammation does not appear to be exacerbated it is certainly maintained, but this is not because of a failure of dexamethasone to suppress inflammation generally, rather a failure to block CNS inflammation.

### Acute neurodegeneration induced by systemic inflammation

Finally, it is of continuing interest that apoptosis of CNS cells is induced by systemic LPS treatment in ME7 animals. The numbers of apoptotic cells reported in the current study are remarkably consistent with those originally observed by us in this experimental paradigm and in that previous study we demonstrated that the apoptotic cells were neuronal by double labelling cells with antibodies against cleaved caspase-3 and neurofilament heavy chain [4]. That this neuronal apoptosis is not blocked by the systemic application of dexamethasone-21-phosphate indicates that systemic cytokines are not essential for this effect. Since CNS production of IL-1β is intact in these animals, we can not rule out a role for CNS IL-1β in the exacerbation of neuronal death previously reported [4]. In an animal model of Parkinson's disease centrally administered LPS exacerbated neuronal death in an IL-1-dependent, iNOS-dependent and glucocorticoid-sensitive fashion [12]. In the current studies, the persistence of neuronal death despite ablation of the systemic cytokine response suggests that these mediators are not necessary for the degenerative effects. It is important to discuss the role of circulating LPS in these effects. Given that we have successfully detected circulating biotinylated LPS in the current study, one might argue that neuronal loss is occurring as a consequence of sepsis/toxic shock. It is well known that severe sepsis has considerable consequences for CNS integrity [49]. However, the LPS doses used in those studies of sepsis in rodents are very considerably higher (20-fold) than those in use here and at 500 μg/kg LPS we have not observed infiltration of inflammatory cells to the brain parenchyma or any significant apoptosis in the normal brain of animals similarly challenged with LPS [4]. Therefore, while we have detected plasma LPS levels in the current study (8.3 ng/ml) we are confident that the effects are not consistent with a state of toxic shock. We have recently shown that Alzheimer's disease patients who suffer relatively mild infections such as respiratory and genitourinary infections show more rapid cognitive decline across a six-month period than those patients who remain infection-free [21]. Whether the doses of LPS used in the current study are pathophysiologically relevant to those infections in AD patients is difficult to state with confidence given the very different sensitivity of mice and humans to endotoxin. However it is relatively clear that in the absence of these low levels of LPS, systemic cytokines such as IL-1β [12] and TNF-α [50] can activate the CNS sufficiently to initiate many of the same cascades in the brain. In this context it is particularly relevant that in a subset of AD patients there was evidence of elevated TNF-α, in the absence of detected infection, and these patients also showed accelerated decline [21]. The finding, here, that circulating cytokines are not necessary for the exacerbation of CNS inflammation and of acute neurotoxicity do not rule out a role for systemic cytokines in producing similar effects: these data show that they are not necessary in the presence of circulating LPS, but may still be sufficient in its absence. The persistence of COX-2 and iNOS responses in the CNS suggests that these mediators may also yet be important in the observed neuronal toxicity.

### Concluding remarks

It is emerging that systemic inflammation has significant deleterious effects on the progression of neurodegenerative disease and understanding the mechanisms by which this occurs is an important priority. It has generally been assumed that cytokines induced by systemic inflammatory stimulation have a key role in the CNS effects of systemic inflammation. While systemic cytokines certainly can achieve a number of CNS effects, the data presented here indicate that these cytokine responses are not essential for the effects on behaviour, CNS inflammation and cell death shown here. Understanding how systemic inflammatory events impact on progression of neurodegenerative disease will have implications for treatment of these patients.

## Competing interests

The authors declare that they have no competing interests.

## Authors' contributions

CM performed the PCR and contributed to data analysis. DS performed biotinylation, ELISA and histological studies and contributed to analysis. CC designed the experiments, performed animal work and histology, contributed to data analysis and wrote the paper. All authors have seen and approved the final version of the manuscript.
